# Effect of original anticonvulsant ortho-fluoro-benzonal on immune cells functional properties at chronic ethanol intoxication

**DOI:** 10.1192/j.eurpsy.2022.2111

**Published:** 2022-09-01

**Authors:** E. Markova, I. Savkin, Т. Shushpanova

**Affiliations:** 1 Federal State Research Institute of Fundamental and Clinical Immunology Russian Academy of Sciences, Neuroimmunology Lab., Novosibirsk, Russian Federation; 2 Mental Health Research Institute of Tomsk National Investigation MedicalCenter of Russian Academy of Sciences, Clinical Psychoneuroimmunology And Neurobiology, Tomsk, Russian Federation

**Keywords:** original anticonvulsant, alchoholism, immune cells

## Abstract

**Introduction:**

GABAa-receptors proved to be the molecular targets of ethanol on immune and nervous cells, potentiating alcohol influence. *ortho*-Fluoro-benzonal is known to be a circular urea derivative and an artificial ligand of GABA/BD-receptor and thus a potential candidate drug for alchoholism treatment.

**Objectives:**

We have shown the alcohol motivation decrease under *ortho*-fluoro-benzonal influence in experiment. The investigation of molecular mechanisms and functional targets of this substance is an important step in understanding of molecular pathogenesis and approaches to managing alcohol addiction.

**Methods:**

Splenocytes from male (CBAxC57Bl/6) F1 mice in a state of alcohol dependence owing to 6-month 10% ethanol exposure were aseptically obtained and cultured in presence of GABA, *ortho*-fluoro-benzonal and mitogens (LPS or concanavalin A). Proliferative activity of immune cells *in vitro* was estimated by means of radioactive ^3^H-thymidine incorporation.

**Results:**

The intact animals’ splenocytes revealed increased spontaneous proliferation, increased T-mitogen stimulated and decreased B-mitogen stimulated proliferation in the presence of *ortho*-fluoro-benzonal. The immune cells from alcoholized animals, demonstrating increased spontaneous proliferative activity and weaken susceptibility to the mitogens, showed normal response patterns, except B-mitogen response case, under *ortho*-fluoro-benzonal inluence. Addition of GABA into the cultures didn’t cancel most positive effects of *ortho*-fluoro-benzonal inluence, proving existence of their GABAaR-independent pathways, mediated by other barbiturate receptors in addition to GABAaR-dependent ones.

**Conclusions:**

Immunomodulating properties of artificial GABA receptor ligand, *ortho*-fluoro-benzonal, *in vitro* has been shown. The compound may correct immune cells dysregulation caused by chronic ethanol exposure, so the original anticonvulsant has promise in the treatment of alcoholism.

**Disclosure:**

The authors have not supplied a conflict-of-interest statement.

